# The Making of Hematopoiesis: Developmental Ancestry and Environmental Nurture

**DOI:** 10.3390/ijms19072122

**Published:** 2018-07-21

**Authors:** Geoffrey Brown, Rhodri Ceredig, Panagiotis Tsapogas

**Affiliations:** 1 Institute of Clinical Sciences, Institute of Immunology and Immunotherapy, College of Medical and Dental Sciences, University of Birmingham, Edgbaston, Birmingham B15 2TT, UK; 2 Discipline of Physiology, College of Medicine & Nursing Health Science, National University of Ireland, Galway, Ireland; rhodri.ceredig@nuigalway.ie; 3 Developmental and Molecular Immunology, Department of Biomedicine, University of Basel, 4058 Basel, Switzerland; panagiotis.tsapogas@unibas.ch

**Keywords:** blood and immune cells, cell lineage, cytokines, fate determination, hematopoiesis, stem cells

## Abstract

Evidence from studies of the behaviour of stem and progenitor cells and of the influence of cytokines on their fate determination, has recently led to a revised view of the process by which hematopoietic stem cells and their progeny give rise to the many different types of blood and immune cells. The new scenario abandons the classical view of a rigidly demarcated lineage tree and replaces it with a much more continuum-like view of the spectrum of fate options open to hematopoietic stem cells and their progeny. This is in contrast to previous lineage diagrams, which envisaged stem cells progressing stepwise through a series of fairly-precisely described intermediate progenitors in order to close down alternative developmental options. Instead, stem and progenitor cells retain some capacity to step sideways and adopt alternative, closely related, fates, even after they have “made a lineage choice.” The stem and progenitor cells are more inherently versatile than previously thought and perhaps sensitive to lineage guidance by environmental cues. Here we examine the evidence that supports these views and reconsider the meaning of cell lineages in the context of a continuum model of stem cell fate determination and environmental modulation.

## 1. Introduction

In 1983, Sulston and colleagues produced a tree map—a series of bifurcations—that delineated the origins of the 671 cells of the newly hatched roundworm *Caenorhabditis elegans* [1]. In this organism, cell lineages and the fates of cells are largely invariant and ancestry therefore determines the end fate of a cell. The apparent rigidity of a tree lineage map ensures tissues develop reliably and consistently. An autonomous lineage programme is also likely to generate the cell types required in a manner that is both efficient and economical. To add to lessons learned from *Caenorhabditis elegans*, there is “unpredictability” in the developmental nature of cells within this organism. Numerous sub-lineages give rise to various populations of motor neurons, rather than these cells descending from the same progenitor in the map. Moreover, when one of the progeny of a terminal division is a motor neuron its “sibling” is unlikely to be a cell of the same type [1].

For over 30 years, tree maps have also been used to describe blood and immune cell development [2] and the development of other tissues (e.g., neuronal-crest-derived cells) [3]. Accordingly, investigations of decision-making by developing cells have tended to examine how they make either-or choices. In the case of hematopoiesis, attention focused on how the progeny of hematopoietic stem cells (HSC) first become common lymphoid progenitors (CLP) and hence generate B cells, T cells and natural killer (NK)/innate lymphoid (ILC) cells, or common myeloid progenitors (CMP), which gives rise to all the other blood cell types [2]. It is then envisaged that later in development progenitors choose, for example, between the neutrophil and monocyte pathways [4] and between the megakaryocyte and erythroid pathways [5]. For years, we have defined hematopoietic cell lineages in terms of just two initial types of oligo-potential cells, CLP and CMP, giving rise to all of the various end-cell types.

**Figure 1:**
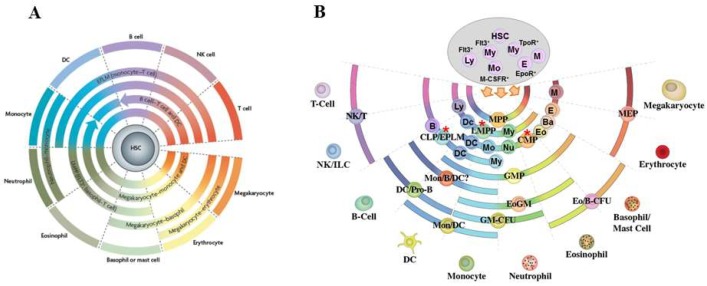
A continuum or pairwise model for hematopoiesis. (**A**) The model envisages a continuum of fates is available to hematopoietic stem cells (HSC), with pairwise relationships between the various cell fates. The pairwise model, replaces a rigid and bifurcating lineage tree with a spectrum of fate options open to HSC. In the “classic” model, HSCs progress stepwise through a series of intermediate progenitors, to close down developmental options in a binary manner. In the new scenario, HSC make an immediate lineage choice from all of the end-cell options. The figure is, with permission, from [6] ^©^Macmillan Magazines Ltd. HSC, hematopoietic stem cell; DC, dendritic cell; NK cell, natural killer cell. (**B**) The partial arcs represent the close relationships between cell lineages that we inferred from the different groups of fates that are available to known intermediary progenitor cells (marked with an asterisk). Investigators initially described Lymphoid-primed multipotent progenitors (LMPP), early progenitors with lymphoid and myeloid potential (EPLM) and common myeloid progenitors (CMP) as homogeneous population of cells. However, they are a mixture of cells with the lineage affiliations shown as cells added to the arcs for LMPP, EPLM and CMP. Affiliations include B lymphocyte (B), DC, monocyte (Mo), eosinophil (Eo), basophil (Ba), erythroid (E) and megakaryocyte (M) [16,17,18,19,20]. HSC are shown to include the following: lymphoid biased cells (Ly) [23,24] that express the fms-like tyrosine kinase 3 receptor (Flt3^+^) [25]; myeloid biased or committed cells (My) [26,27] that express Flt3^+^ [25] and/or the receptor for thrombopoietin (TpoR^+^) [28]; cells committed to the erythroid pathway [19] and affiliated as to expression of the receptor for erythropoietin (EpoR^+^) [25,29]; and cells that express the receptor for macrophage colony stimulating factor (M-CSFR^+^) and monocyte-affiliated (Mo) [25,30]. CLP, common lymphoid progenitor; CMP, common myeloid progenitor; DC/Pro-B, dendritic cell and B lymphocyte progenitor; Eo/B-CFU, eosinophil and basophil progenitor; EPLM, early progenitor with lymphoid and myeloid potential; GMP, granulocyte and macrophage progenitor; LMPP, lymphoid-primed multipotent progenitor; MEP, megakaryocyte and erythrocyte progenitor; Mon/B/DC?, monocyte, B lymphocyte and dendritic cell? progenitor; Mon/DC, monocyte and dendritic cell progenitor, NK/T, natural killer cell and T lymphocyte progenitor and NK/ILC, natural killer cell and innate lymphoid cell progenitor.

In recent years, descriptions of the architecture of haematopoiesis have been moving away from the idea of HSC development progressing via: a) a series of intermediate oligo-potent progenitors; and b) these cells making a series of binary choices that stepwise restrict lineage options, ultimately to one final fate. In a new scenario, the suggestion is that HSC can make an immediate lineage choice from a continuum that encompasses all of the end-cell options. Our pairwise model, proposed in 2009, (Figure 1A) [6], does not prescribe an invariant route to each end-cell type via definitive intermediate progenitors. It presumes, quite simply, that all of the options are open to HSCs. This is in keeping with decision-making, regarding lineage affiliation, at the level of the HSC and very different from HSC making an immediate and binary choice, for example, between the myeloid and lymphoid fates. In support of this new viewpoint is the recent finding that HSC in mice can undergo restriction to the myeloid, megakaryocyte/erythroid and megakaryocyte pathways without dividing or entering S phase of the cells cycle [7]. Previously, we showed that the human promyeloid cell line HL60 makes a choice between the neutrophil and monocyte fates without dividing and when held in the G_1_ and S phases of the cell cycle [8,9]. Cell fate decisions are therefore uncoupled from cell division and the mechanisms that control fate determination/differentiation and proliferation are at least partly independent. An inverse relation between cell cycle and differentiation can actually occur, as has been shown in the case of the transcription factor PU.1 in myeloid differentiation. Increased PU.1 levels can lead to prolongation of the cell cycle resulting in further accumulation of PU.1 and thus commitment of progenitors to the macrophage lineage [10].

The concept of a continuum of developmental options is important and applies to the description of various changes to the status of cells. A longstanding viewpoint is that embryonic stem cells make a binary choice between pluripotency and specification to a germ cell layer. However, the stem cell compartment of human embryonic stem cell cultures is significantly heterogeneous and Hough and colleagues have argued that a continuum of states spans pluripotency and lineage commitment of these cells [11]. Moreover, the existence of self-renewal as a canonical state is debatable: cells progressively decrease their likelihood of self-renewal as the expression of stem cell- and pluripotency-related genes decreases and that of genes encoding differentiation attributes increases [11]. Epiblast cells of the early mammalian embryo are able to make mesoderm, endoderm or ectoderm fate decisions and as development progresses they mature through a continuum of lineage potency states [12]. Additionally, a continuum best describes the progression of cell differentiation. For example, early studies revealed gradual changes in the expression of a large number of surface antigens during the different stages of neutrophil development [13]. Similarly, maturation changes occur along a continuum during erythropoiesis [14]. To return to *Caenorhabditis elegans*, a continuum framework has been used to model epithelial morphogenesis and elongation of the embryo to a worm [15].

The pairwise model also emphasizes that there are particular nearest neighbour relationships between the various cell lineages. The known sets of fates of cells termed lymphoid-primed multipotent progenitors (LMPP), early progenitors with lymphoid and myeloid potential (EPLM) and common myeloid progenitors (CMP) and of other downstream progenitors (see Figure 1A,B), guided the construction of the relationships of progenitors in the pairwise model. However, recent genotypic studies have revealed that LMPP, EPLM and CMP although phenotypically relatively homogenous are not homogeneous populations and instead are mixtures of cells with different lineage affiliations [16,17,18,19,20]. We have examined the affiliations of EPLM, which were originally described as a population of cells that could generate B and T lymphocytes, NK cells, dendritic cells (DC) and macrophages but that lacked megakaryocyte and erythroid potentials [21]. Thus, by using the surface markers Ly6D, SiglecH and CD11c and by RNA sequencing single cells, we separated EPLM into four subpopulations each with lineage biases. We divided the most primitive EPLM, lacking these markers, into cells that already have myeloid, DC or lymphoid signatures [16]. Likewise, Naik and colleagues have similarly divided LMPP into lymphoid-, myeloid- and DC lineage-biased cell populations [18]. Hoppe and colleagues used RNA expression data to classify CMP into granulocyte/monocyte and megakaryocyte/erythroid progenitors [17] and Paul and colleagues have assigned bone marrow progenitors into seven groups with transcriptional characteristics of neutrophils, basophils, eosinophils, monocytes, DC, erythrocytes and megakaryocytes [20]. Figure 1B shows the sub-populations of LMPP, EPLM and CMP as cells added to their arcs of potentials. The particular close relationships between cell lineages remain the same as lineages adjacent to one another share the usage of transcription factors and cell responsiveness to promiscuous cytokines [6,22]. This fits with the notion of visualising cell specification as a continuum and infers that adjacent elements are less different from each other whereas the extremes are quite distinct.

There is good evidence to support the view that the lineage affiliations seen in recent investigations of LMPP, EPLM and CMP become apparent earlier during development, even as early as in HSC. In 2010, Ichi and colleagues reported that the transcriptional profiles of individual HSCs show considerable variation and some HSCs are poor precursors of lymphocytes whilst others generate a balanced output of cell types [31]. Subsequently, investigators examined the progeny of single cells transferred into irradiated mice and described myeloid- and lymphoid-biased mouse HSC [24,26,32,33]. Notta and colleagues have shown that the progenitor cell compartment of human bone marrow is a mixture of cells with uni-potent myeloid or erythroid potential alongside multipotent cells [19]. The Jacobsen group described a sub-set of murine HSC with a bias towards platelets and myeloid cells, requiring thrombopoietin for their maintenance [28]. Similarly, sub-sets of murine HSC express the receptor for macrophage colony-stimulating factor (M-CSF), mRNA for the erythropoietin (Epo) receptor [25,29,30] and the fms-like tyrosine kinase 3 (Flt3) receptor, for Flt3 ligand (Flt3L) [25]. The selective expression of these receptors is suggestive of a predisposition and/or affiliation of some HSC to a lineage because the corresponding cytokines (M-CSF, Epo and Flt3L) can instruct end-cell fate ([30,34] and see later).

## 2. The Mature End-Cell Populations Are Not Homogeneous

**Figure 2:**
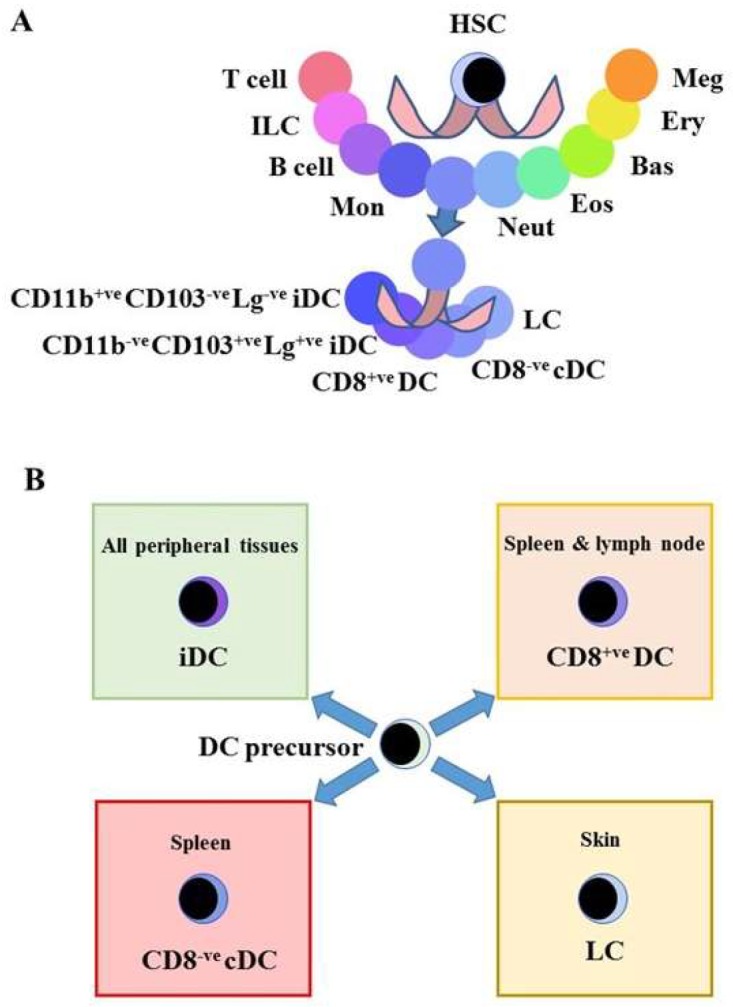
Heterogeneity of dendritic cells. (**A**) Surface markers are used to describe the various end-cell DCs and their nature is a matter of gradations. (**B**) Where each population resides is important to their function within the immune system and the local environment may determine the fate of DC precursors [41]. HSC, hematopoietic stem cell, ILC, innate lymphoid cell, Mon, monocyte; Neut, neutrophil; Eos, eosinophil, Bas, basophil; Ery, erythrocyte; Meg, megakaryocyte, DC, dendritic cells, iDC, interdigitating dendritic cell; LC, Langerhans cells; Lg, Langerin.

The variety of end-cell types is more complex than is usually shown in models. For example, the DC family includes Langerhans cells (LC), two types of interstitial DC (iDC) [35,36], CD8^+^ DC [37] and CD8^−^ conventional DC (cDC) [37] (Figure 2A). The delineation of these DC sub-populations depends on their precise functions. They include antigen transport, immune regulation and the control of infections e.g., yeast. In addition, there are type 1 interferon-producing plasmacytoid pDC [38] and monocytes that are a form of pre-DC [39] and both are classified within mononuclear phagocytes (see later). The local environments in which DC precursors reside may be important for their fate specification (Figure 2). Satpathy and colleagues have highlighted the importance of various cytokines to DC development, including whether their actions within distinct stromal niches influence fate choice [40].

There is heterogeneity among cells of the mononuclear phagocyte system [42]. Human monocyte sub-populations are discriminated on the basis of CD14 and CD16 expression and those in mice by the use of markers such as F4/80, Ly6C (Gr-1), CD11b, CD43 and the chemokine receptor CX3CR1 [43]. Following trans-endothelial migration, monocytes enter the tissues where they give rise to macrophages. There is debate about how best to distinguish macrophages from DCs: is there any real difference between the capacity of some macrophages and DCs to present antigens [44]? Live imaging studies have not provided clear evidence for a distinction [45]. Additionally, a population of cutaneous Langerin^+ve^ DC develops from blood monocytes, rather than from Langerhans cells [35,46]. 

The markers used to define macrophage sub-sets include the macrophage scavenger receptors (CD36, CD14, signal-regulatory protein family (SIRP)), Toll-like receptors, various integrins, epidermal growth factor-seven transmembrane (EGF-TM7) proteins, other immunoglobulin superfamily receptors (Siglecs) and multiple C-type lectins. Our perception of the assortment of these markers is that it is apparently random [47]. The use of quantitative flow cytometry (FC) profiles reveals a spectrum of phenotypic variability, rather than precisely defined sub-sets of cells [42]. Some of this phenotype noise may be due to stochastic fluctuations in the levels of expression of transcription factors. Even so, the different macrophage sub-sets, as defined by different combinations of markers, provide distinct signals to T cell sub-sets and are therefore important physiologically. 

Macrophages encounter multiple signals in the various tissues that are important for shaping their phenotype [48]. For example, macrophages that reside in the lung and in the peritoneum have distinct gene expression profiles and chromatin landscapes. Lavin and colleagues transferred macrophages that had been isolated from the mouse peritoneum into the alveolar cavity, whereupon they upregulated the macrophage-specific genes that typify lung macrophages and downregulated the peritoneal macrophage-specific genes. Expression profiling confirmed this transition: the transferred cells resembled lung macrophages more than peritoneal macrophages. So, the originally peritoneal macrophages had adapted to their new environment [49]. Further evidence of macrophage adaptability is that CD8^+ve^ and CD8^−ve^ antigen-presenting cells in the spleen that arise from the same progenitor are interconvertible [50]. These findings both highlight the adaptability of differentiated cells and show that the microenvironment can influence cell specialization. Signals from the tissue environment may modulate the chromatin landscape that, in turn, prescribes a tissue-related gene expression pattern within macrophages.

## 3. Some End-Cells Are Interconvertible

CD4^+ve^ effector cells include T helper 1 cells (Th1), T helper 2 cells (Th2), interleukin (IL) 17-producing T helper cells (Th17), follicular T helper cells (Tfh) and regulatory T cells (Treg). These sub-types have different functions. They include the control of bacterial, helminth and fungal infections and B cell maturation in germinal centres and the suppression of immune responses. CD4^+ve^ effector cells produce many different cytokines, including IL-2, IL-3, IL-4, IL-13, IL-17, IL-21 and granulocyte-macrophage colony-stimulating factor (GM-CSF) and to a lesser extent interferon g (IFNγ) [51]. The nature of the cytokines produced is indicative of the type of CD4^+ve^ helper cell. CD4^+ve^ cell types arise in multiple environments and the prevailing cytokine environment is important for their differentiation. For example, IL-12 is required for Th1 cell differentiation and IFNγ amplifies this process, whereas IL-4 drives the differentiation of Th2 cells [52].

**Figure 3:**
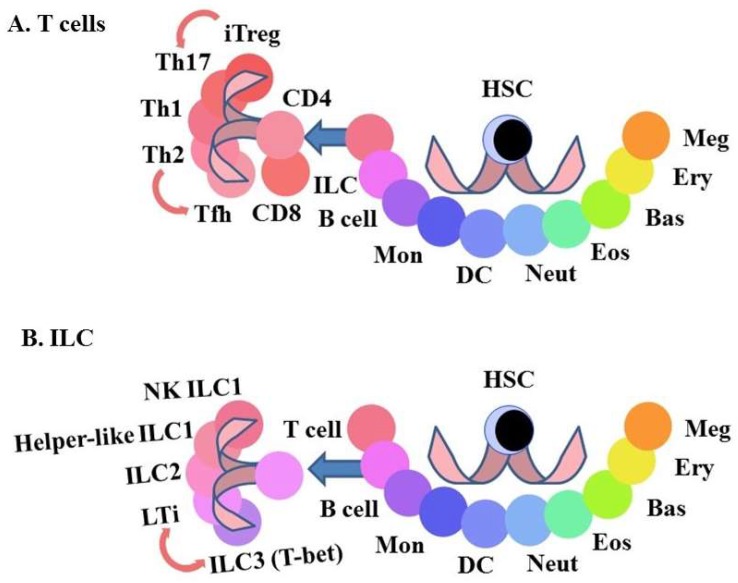
The heterogeneity and interconversion of CD4^+ve^ helper T lymphocytes and innate lymphoid cells. CD4^+ve^ effector cells and innate lymphoid (ILC) cells include various sub-populations. For both of these types of cells, the characteristics of some of the mature effector cells are interconvertible (**A**) The arrows connecting the sub-types of CD4 T cells show interconversions. (**B**) The arrows connecting the sub-types of ILC show interconversions. HSC, hematopoietic stem cell, iTreg, induced-regulatory T cells; Th17, Interleukin 17 producing T helper cells, Th1, T helper 1 cells; Th2, T helper 2 cells, Tfh, follicular helper T cells; NK, natural killer cell, Mon, monocyte; Neut, neutrophil; Eos, eosinophil, Bas, basophil; Ery, erythrocyte; Meg, megakaryocyte, DC, dendritic cells.

The characteristics of some of the mature CD4^+^ effector cells are interconvertible (Figure 3). IL-21 is produced by Th2 cells [53] and TFh cells [54] and Th2 cells can give rise to Tfh cells [55]. Induced regulatory T cells (iTreg) can convert to pro-inflammatory Th17 [56] and when transferred into T cell-deficient CD3^−/−^ or Rag2^−/−^ mice respectively give rise to Tfh cells in Peyer’s patches [57] and Th2 cells in the spleen [58]. Memory Th2 cells convert in vitro to iTreg, in response to treatment with transforming growth factor β (TGF-β) and blockade of IFNγ and IL-4 signalling [59]. In all of these examples, the switching between the different types of CD4^+^ cells requires an external set of signals, suggesting that environmental signals may underlie the adaptability of CD4^+^ cells to the type of the immune response required. Eizenberg-Magar and colleagues have constructed mathematical models that predict how different cytokine inputs to CD4^+^ T cells determine their differentiation state [60].

The sub-types of ILC have natural killer and helper-like functions that are important as a first line defence against pathogens, the genesis of lymphoid organs and tissue modelling [61]. There are three main groups of ILC. Group 1 includes natural killer cells and helper-like ILC and groups 2 and 3 are helper-like. The the expression of transcription factors defines two populations within groups 1 and 2. The developmental relationship between the various ILC populations is presently unclear and their characteristics are interconvertible (Figure 3). Expression of master regulators of transcription, surface receptors and the ILs produced define group 3 sub-types. There are cells that either: (1) express the transcription factor retinoid-related orphan receptor γt (RORγt), CD4 and the cytokine receptor CCR6 at their surface and produce IL-17 and IL-22 (lymphoid tissue inducer (LTi)-like ILC3) or (2) express the transcription factor T-bet, the NK receptors NKp46 and NK1.1 at their surface and produce IFNγ and TNFα. However, LTi-like ILC3 (RORγt^+^, CCR6^+^) cells express T-bet when exposed in vitro to a Notch stimulus [62]. There may be a true bi-directional plasticity of ILC3 as NKp46^+^ ILC3 may downregulate NKp46 in vivo giving rise to NKp46^−^ ILC3 [63]. The relative levels of expression of RORγt and T-bet may determine the effector functions of group 3 ILC3 and underlie the plasticity of the phenotype of these cells. However, there is controversy on this matter as the subsets of group 3 ILC3 may be separate lineages that develop from different progenitors [64]. To add to the plasticity of ILC, mouse studies have shown that ILC2 that reside in the lung become ILC1 in response to infection by influenza virus and *Staphylococcus aureus* and by cigarette smoke [65].

## 4. How Might We Classify the Types of Cells?

One purpose of classifying blood cells is to aid the understanding of their development: we have no hope of understanding cell diversification without categorizing a cell’s identity. The conventional use of the term cell lineage and cell type, refers to the developmental history of a cell. For example, a progenitor cell that is committed to the B lymphocyte developmental pathway gives rise to cells we denote as a B lymphocyte. However, ancestry does not always resolve cell identity where there is inconsistency between the attribution of cells to a lineage and classification with regard to a phenotype. For cells viewed collectively as ILCs, there are two separate origins; a progenitor that gives rise to the NK precursor and NK cells and another for all the helper-like ILC [61]. Similarly, it is not clear to what extent there are separate progenitors for the different DCs. They appear to arise from two separate -lymphoid and monocytic- origins but the surface phenotypes and gene transcription profiles of DCs derived in vitro from purified CLPs or purified CMPs are indistinguishable [66]. A Common Dendritic cell Progenitor (CDP) with the ability to give rise to both cDC and pDC has been identified [67,68]. Several other phenotypically distinct cells have been proposed as progenitors of different DC sub-populations [67,69,70,71]. However, it appears that multiple developmental pathways are at play in generating the different DCs, and, in some cases, they converge into phenotypically homogeneous but transcriptionally and functionally distinct mature DC [72,73]. The delineation of cell type with regard to ancestry is also confounded if we accept that HSCs predispose to a lineage by expressing, for example, the receptor for M-CSF but might step sideways and adopt a different trajectory. 

In the case of the mature immune cells, an answer to the problem of their classification, their attributes or conversely the absence of a characteristic(s), is the unique function of each type of cell. In other words, members of a cell type serve a function that is different from members of another cell type. However, immune cell types can share functional attributes that confounds ascribing cell identity on this basis and blurs the boundaries between cell lineages. A cytotoxic capacity brings together some T cells and some ILC, whereas macrophages, DC and B cells can phagocytose, pinocytose, process and present foreign antigens. Additionally, cells of the immune system cooperated to perform their role and it is therefore not too surprising that different types of cells share, for example, the chemokine receptors that dictate the location of cells to a particular environment and the cytokine receptors for survival. 

## 5. What Are the Differences between Types of Cells?

So, what are features that allow us to specify a population of immune cells? Distinguishing one cell type from another is in essence a matter of how many phenotypic markers we use to define a cell type. The use of two surface markers can clearly differentiate one type of cell from another. However, and as mentioned above, use of additional surface markers reveals substantial heterogeneity regarding mononuclear phagocytes and investigators must rely on their judgment as to how best to classify cell populations. Traditionally, the basis for the identification of early progenitors, in particular, is the use of a limited number of cell surface markers, which appear in many cases to have a graded, rather than discontinuous expression pattern. Examples of graded expression are the markers used for FC-based identification of Lineage-negative, CD117^+^ (kit), Sca-1^+^ cells (LSK), LMPP and CLP populations.

**Figure 4:**
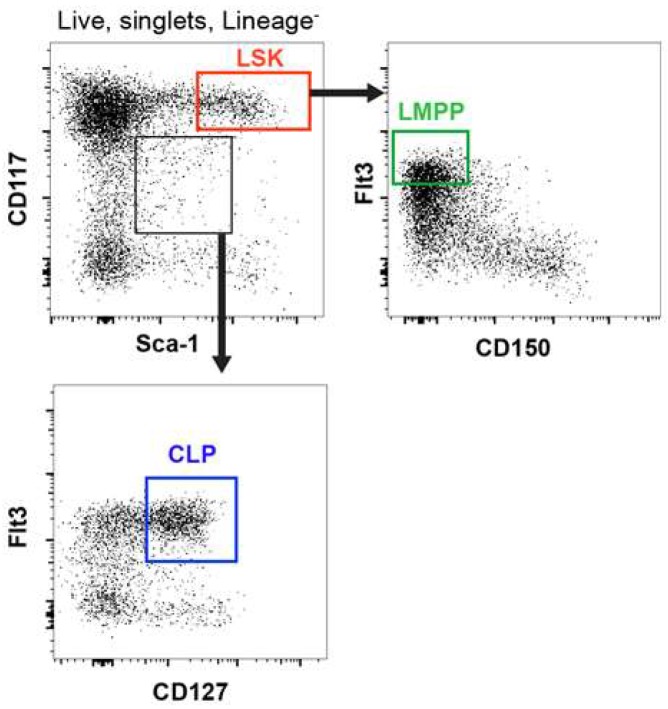
Markers used to identify hematopoietic progenitors display graded, rather than discontinuous, expression. CD117, Sca-1 and Flt3 show a continuous expression making the identification of cells with “high” or “intermediate” expression of markers almost arbitrary. Figure shows a typical gating strategy for the identification of LSK (red), LMPP (green) and CLP (blue) progenitor populations based on the expression of CD117, Sca-1, Flt3 and CD127 (IL-7Rα). LSK are identified as Lineage^-^CD117^high^Sca-1^high^ [2] and LMPP as the LSK with the highest expression of Flt3 [74]. CLP are gated as Lineage^-^CD117^int^Sca-1^int^Flt3^+^CD127^+^ [75,76]. LSK: Lineage-negative, Sca-1-positive, kit-positive cells; LMPP: Lymphoid-primed Multi-Potent Progenitors; CLP: Common Lymphoid Progenitors.

CD117, Sca-1 and Flt3 show a graded, continuous expression, thus making the identification and isolation of cells with “high” or “intermediate” expression of markers almost arbitrary and dependent on the staining prowess and judgement of the investigator (Figure 4). This problem becomes even larger for genetically modified mice, where it might prove difficult to distinguish between true changes in the proportions of gated cells (representing progenitor stages) and any potential alterations, caused by the genetic modification to the mice, in the expression level of markers that are used to “ring-fence” these cells. Modern and comprehensive analyses, particularly of single cells, have revised the approach to categorize cell types. Tools currently available for the examination of cellular heterogeneity include global gene expression analysis and mass cytometry where the number of individual surface markers analysed is extremely large. This leads to an even more detailed consideration of how finely to draw lines between different cell types and perhaps this becomes arbitrary for some of the different types of immune cells.

Regarding the use of global gene expression analyses, the conceptual problem is how fine should the criteria be that we use to distinguish one type of cell from another? A caveat is that it is impossible to be certain that two cells that, using a set of phenotypic attributes are ring-fenced as identical, are identical genotypically [77]. A simple reason is that cells of any given end-cell type are not all interacting with just one environment: some will be inside a particular environment and some left outside. Even within an environment, a graded input of signals might well lead to a graded output regarding the level of acquisition/stabilization of a phenotype. Moreover, a population of cells, even if clonal, is heterogeneous. Chang and colleagues have shown that cells of cloned populations of mouse hematopoietic progenitors express different levels of Sca-1. This relates to a state that fluctuates but is meaningful because cells that express extremely high or low levels of Sca-1 have distinct transcriptomes and different leanings towards the myeloid or erythroid lineage [78]. Heterogeneity of cloned cells is also the case for neuronal stem cells whereby in vitro subsets have distinct developmental commitments [79]. The heterogeneity of cloned stem cells presumably reflects the inherent versatility of multipotent cells. Of particular interest to lineage-predisposition of these cells are the events that regulate the expression of transcription factors. It would thus seem that none of the classically accepted features alone is reliable enough to unequivocally define a particular lineage or cell type. Rather, we should consider a combination of several attributes, including morphology, phenotype, function (for mature cell types), genetic signature and developmental ancestry.

For years, the issue of delineation has applied for how to best classify organisms into species, such as Darwin’s many finches. One solution is that each species of finch breeds true. In a similar vein, whether cells can or cannot readily, even when “pushed” in vivo or in vitro, interconvert provides a way of resolving the delineation of cell types. The matter of definition of cell type and lineage, if we view this a synonymous, is simple if both refer to a cell that has progressed along a pathway to a stage of development and a phenotype that is irreversible. For example, and to the best of our knowledge, a cell, including its phenotypically identifiable progeny in the bone marrow, destined to generate mature B cells does not normally give rise to a mature T cell. Interestingly, the importance of the extent of developmental progression to irreversible lineage affiliation is exemplified by the finding that the Double Negative 2 T cell progenitor population that have already progressed some way along the T cell pathway can still give rise to NK and myeloid cells when “pushed” using the right conditions [21,80]. Nevertheless, and by applying the rule of irreversibility of phenotype, we would view the different ILC and CD4^+^ T cells that can interchange/reverse mature fates under the right conditions as sub-types of cells belonging to the ILC and CD4^+^ T cell lineages respectively, rather than as separate lineages. As to their taxonomy, we might view their differentiation and end phenotype as a much more flexible and continuous process that circumvents the need to draw overly strict boundaries. In this case, there is a continuum of intermediate states between cell sub-types.

## 6. Does the Environment That Cells Reside in Instruct Cell Fate?

As mentioned above, cells and their genes, do interact with and respond to their environment. The environmental history of a cell is therefore important to the specification of phenotype. Whilst for cell identity we have attached prime importance to developmental history, namely who begat whom, this might merely serve to keep offspring within a particular nurturing environment. In some instances, we might have mistakenly read environmental nurture as developmental history.

Some hematopoietic cytokines are important for shaping hematopoiesis. Generally, we accept that they are essential for the survival and proliferation of hematopoietic cells at all stages of their development. However, in some cases they are also important for the differentiation of hematopoietic lineages. Thus, their function as merely survival and/or proliferation agents (permissive role) or inducers of differentiation (instructive role) has been a matter of debate for long time [81,82,83]. Several investigations have provided evidence in support of both modes of cytokine action, with the data in some cases being contradictory (reviewed in: [84,85,86]). Genetic deletion of cytokines or their receptors has resulted in a reduced production of immune cells whose generation is subject to regulation by the cytokine in question but even though the disruption in developmental output can be quite severe, it has never led to the complete absence of the lineage. This indicates either a permissive role of these cytokines or some level of redundancy, with other cytokines compensating for the absent instructive signal. Furthermore, over-expression of anti-apoptotic signals, such as B-cell lymphoma 2 (Bcl2), in order to provide a strong survival signal to cells, has been on some occasions sufficient to rescue the affected lineage, suggesting a permissive cytokine function. Thus, whilst the absence of IL-7 signalling severely disrupts T cell development, Bcl2 over-expression can significantly rescue T cell output in these mice [87,88,89], suggesting a survival role of IL-7 in early T cell development. Similarly, the B cell defect observed in mice lacking Stat5 (the crucial signalling mediator for IL-7) can be fully recovered by Bcl2 over-expression [90], while sustained and increased Flt3L levels can rescue B cell development in *Il7^-/-^* mice [91], thus demonstrating the permissive role of IL-7 in both T and B cell development. Moreover, early in vivo studies with chimeric receptors provided further evidence for a permissive role of hematopoietic cytokines. The replacement of the intra-cellular part of the TPO receptor (mpl) with that of G-CSF [92] or the signalling domain of G-CSF with that of Epo-R [93] did not result in any lineage skewing, as would be expected regarding an instructive role of the corresponding cytokines.

Other transgenic approaches, mainly by ectopic expression of cytokine receptors, have nevertheless suggested instruction of lineage fate by cytokines. Thus, expression of M-CSF receptor in multi-potent hematopoietic cell lines resulted in skewing of their developmental output [94,95], while ectopic GM-CSF receptor expression on CLP or pro-T cells increased their myeloid differentiation potential [96,97,98]. Similarly, Flt3 expression in MEP up-regulated myeloid-specific transcription factors and promoted their differentiation towards granulocyte/macrophage lineages [99]. More recently, investigators have provided in vitro and in vivo evidence for an instructive role of cytokines in lineage fate. Our studies support an instructive role of Flt3L at an early stage of hematopoiesis [34]. Transgenic mice over-expressing Flt3L ubiquitously are anemic and thrombocytopenic. Investigation of the progenitor cell populations in these mice revealed that Flt3L acts to skew hematopoiesis towards the myeloid and lymphoid lineages, as there is a lack of generation of erythroid and megakaryocyte progenitors. The cells diverted lack expression of cell lineage markers (Lin^-^) but express the Sca-1 antigen and the receptor for the stem cell factor CD117 or c-kit and are termed LSK. Likewise, increasing the level of Epo in vivo, by transgenesis, has revealed an instructive role for this cytokine in erythropoiesis [29]. In 1982, Metcalf and Burgess provided evidence supporting the view that G- and GM-CSF instruct bi-potent granulocyte-macrophage colony forming cells in their choice between granulocyte and macrophage pathways respectively [4]. Rieger and colleagues added to these finding by showing that G- and M-CSF instruct granulocyte and macrophage progenitors to follow each of the pathways respectively [100].

Overall, there is evidence to support the view that some of the hematopoietic cytokines can instruct cell fate and therefore guide the generation of a particular type(s) of cell as required. The effect of cytokines seems to be cell-context dependent, highlighting the interplay between extra-cellular signals from the environment and intra-cellular fate-determining factors, such as transcription factors and the epigenetic landscape [86]. Additionally, some level of promiscuity in the use of cytokines by hematopoietic cells makes the identification of their specific mode of action challenging. In that context, studies at the single cell are necessary. As evident from the data discussed above, most of the experimental evidence for an instructive role of hematopoietic cytokines comes either from in vivo over-expression of cytokines and/or their receptors or from in vitro culture systems. It is conceivable that, depending on the strength of the signal, a particular cytokine may act to provide a signal for survival, to proliferate or to instruct lineage choice. The response elicited within a cell, as to which of the three outcomes is undertaken, would accord with an increasing level of signal intensity. Thus, in order to demonstrate their instructive role, it is necessary to use a high level of cytokine in in vitro experiments. A graded response of cells to cytokines may be important for steady state versus hematopoiesis under stress conditions, such as infection, whereby the different requirements are survival/expansion versus survival/expansion coupled to a rapid and emergency, diversion of cells towards a required end cell type [101]. Recent evidence to support this viewpoint is that Singh and colleagues have observed that during chronic erythroid stress, in Epo transgenic mice, HSC exhibit a vastly committed erythroid progenitor profile together with enhanced cell division [102].

This makes physiological sense when one considers that the hematopoietic system is extremely flexible, responding rapidly to situations of stress, infection and blood loss. Whilst there is debate as to how this operates, it is important to bear in mind that cells we identify as HSC that can reconstitute the entire hematopoietic/immune system long term (LT-HSC) express the receptors for the proposed instructive cytokines. These include the receptors for Epo, M-CSF and Flt3. Around 19% of LT-HSC express the receptor for M-CSF on their cell surface and ~5% express cell surface Flt3, with ~12% expressing *Flt3* mRNA. Around 13% express mRNA for the Epo receptor and the level of surface protein expression is unknown due to the lack of a suitable antibody reagent. These are essentially sub-populations of LT-HSC [25]. We might assume that engagement of the cytokine with the receptor provides a signal for survival and proliferation. We do not know whether the presence of the appropriate cytokine instructs LT-HSC to adopt either a macrophage, myeloid or erythroid fate. 

## 7. The Events That Shape Cell Identity

**Figure 5:**
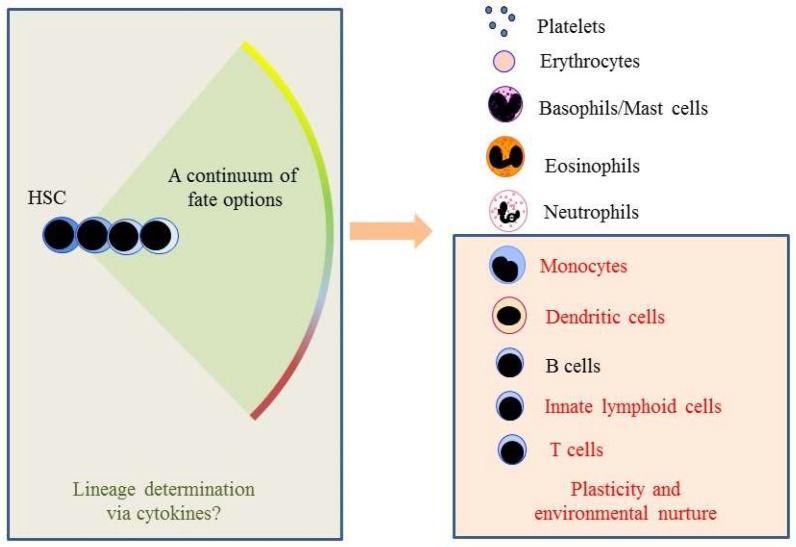
The developmental versus environmental history of cell types. A continuum of options is open to HSC and they are able to choose a fate without having to progress stepwise through a series of intermediate progenitors in order to close down developmental options. There is good evidence to support the viewpoint that environmental nurture is important to the generation of diversity of some of the mature cell types.

It is reasonable to conclude that the nature of immune cell diversity is not merely a matter of affiliation to an ancestral pathway but that environmental nurture is important. A mixture of (i) predisposition towards/affiliation of HSC to a lineage and (ii) nurture at a later stage of development, by environmental cues, appear to govern the end-fate of a cell (Figure 5). This perhaps explains the many sub-types of a number of different immune cells. Nurture is also important for HSC. It is likely that the localization of HSC within diverse and supportive bone marrow niches and their exposure to different development growth factors and morphogen gradients plays an important role in driving their heterogeneity [103]. 

Waddington’s epigenetic landscape has provided a longstanding model for the general nature of the events that shape cell identity [104]. Cells roll down bifurcating valleys and once a cell has chosen a fate there are ridges that help maintain this fate. Changes to the epigenome dictate the hills and valleys that exclude other fates and which the microenvironment might impose. Ferrell has proposed an alternative landscape whereby at the start there are numerous valleys and ridges, commitment to a fate relates to the disappearance of some of the valleys and ridges and new valleys and ridges arise from cell-cell competition [105]. Sieweke examines a landscape that he likens to a small group of Pacific islands—Captain Cook’s Islands—in the middle of an ocean. These can be reached from many directions such that the routes to a fate are flexible [106]. Waddington’s landscape and the alternative landscape both involve stepwise bifurcations but there does not seem to be the need for HSC to undergo this progressive restriction. These models and a “small group of islands” model mean that there are close relations between particular cell lineages, as envisaged in the pairwise model. A “small groups of islands” model is more in keeping with the pairwise model except that each island can be approached separately rather than by Captain Cook’s route from island to island and from east to west.

## 8. Implications for Leukemia

Normal HSCs and their immediate progeny are versatile in their choice of developmental pathway. If we accept that HSC can commit themselves directly to one pathway, we might presume that each pathway is equally available. Versatility is also the case for the progeny of HSC. The colonies observed for the progeny of HSC, particularly multipotent HPC, grown in semi-solid agar or methylcellulose medium contain many different cell types. We have argued that these findings are misleading as to how HSC and HPC behave in vivo, because single cells dispersed in agar are not in their normal social environment and lack the influence of niches, the extracellular matrix and the appropriate cytokines [101]. Nevertheless, the findings from colony assays support the notion of inherent versatility of HPC and perhaps indicate that environmental influences in vivo are important for guiding and/or narrowing the trajectories of HSC. HSC are versatile in the pairwise model, because they can step sideways to adopt alternative, closely related, fates, even after they have “made a lineage choice.” The making of the architecture of leukemia and lymphoma, is very different because the lineage output of the transformed counterparts is restricted to a particular pathway [107,108,109]. In contrast to the flexibility of HSC, the transformed counterpart essentially dumps cells down a particular pathway. The rationale for this and perhaps the cardinal aspect of cell transformation in cancer is that a genetic insult fixates the epigenome to a lineage. Sanchez-Garcia has proposed that the contribution of oncogenes to leukemia is via epigenetic priming of the cells that initiate leukemia and the initial oncogenic insult(s) is/are then dispensable for tumour progression and maintenance [109]. In other words, there appears to be an insult-wired block to versatility in the leukemia stem cell. 

## 9. Concluding Remarks

In the pairwise model, HSC can immediately predispose towards or affiliate to a single lineage, yet retain the ability to divert to an adjacent lineage. We might view the adjacent options as easily accessible or latent in some way. In this case, predisposed or affiliated HSC are essentially progressively differentiating towards an outcome, including narrowing their trajectory from the continuum of options. In the original diagram for the pairwise model, we showed DC, monocytes and NK cells as a single population. Each of these cell types is clearly a heterogeneous population and, therefore, it seems that some cells have a lifelong capacity to differentiate and diversify. Questions that are perhaps more important than lineage affiliation are (i) what channels a cell towards a particular phenotype and (ii) how does a cell stabilize a pattern of attributes, some of which are shared by different cell types. Perhaps the signals they receive from the environment in which they reside, that “mark” the epigenome, are as important as developmental history. Our view of the architecture of haematopoiesis has important implications to our understanding of the initiation of leukaemia. Whereas HSC are versatile, an insult(s) appears to wire the epigenome of HSC regarding lineage choice to a fixed leukaemia cell fate [109].
